# Drug-Resistant Genotypes and Multi-Clonality in *Plasmodium falciparum* Analysed by Direct Genome Sequencing from Peripheral Blood of Malaria Patients

**DOI:** 10.1371/journal.pone.0023204

**Published:** 2011-08-11

**Authors:** Timothy Robinson, Susana G. Campino, Sarah Auburn, Samuel A. Assefa, Spencer D. Polley, Magnus Manske, Bronwyn MacInnis, Kirk A. Rockett, Gareth L. Maslen, Mandy Sanders, Michael A. Quail, Peter L. Chiodini, Dominic P. Kwiatkowski, Taane G. Clark, Colin J. Sutherland

**Affiliations:** 1 Wellcome Trust Centre for Human Genetics, University of Oxford, Oxford, United Kingdom; 2 Wellcome Trust Sanger Institute, Hinxton, United Kingdom; 3 Global Health Division, Menzies School of Health Research, Charles Darwin University, Darwin, Australia; 4 Department of Clinical Parasitology, Hospital for Tropical Diseases, London, United Kingdom; 5 Faculties of Infectious and Tropical Diseases and Epidemiology and Population Health, London School of Hygiene & Tropical Medicine, London, United Kingdom; Johns Hopkins School of Public Health, United States of America

## Abstract

Naturally acquired blood-stage infections of the malaria parasite *Plasmodium falciparum* typically harbour multiple haploid clones. The apparent number of clones observed in any single infection depends on the diversity of the polymorphic markers used for the analysis, and the relative abundance of rare clones, which frequently fail to be detected among PCR products derived from numerically dominant clones. However, minority clones are of clinical interest as they may harbour genes conferring drug resistance, leading to enhanced survival after treatment and the possibility of subsequent therapeutic failure. We deployed new generation sequencing to derive genome data for five non-propagated parasite isolates taken directly from 4 different patients treated for clinical malaria in a UK hospital. Analysis of depth of coverage and length of sequence intervals between paired reads identified both previously described and novel gene deletions and amplifications. Full-length sequence data was extracted for 6 loci considered to be under selection by antimalarial drugs, and both known and previously unknown amino acid substitutions were identified. Full mitochondrial genomes were extracted from the sequencing data for each isolate, and these are compared against a panel of polymorphic sites derived from published or unpublished but publicly available data. Finally, genome-wide analysis of clone multiplicity was performed, and the number of infecting parasite clones estimated for each isolate. Each patient harboured at least 3 clones of *P. falciparum* by this analysis, consistent with results obtained with conventional PCR analysis of polymorphic merozoite antigen loci. We conclude that genome sequencing of peripheral blood *P. falciparum* taken directly from malaria patients provides high quality data useful for drug resistance studies, genomic structural analyses and population genetics, and also robustly represents clonal multiplicity.

## Introduction

Naturally acquired blood-stage infections of the malaria parasite *Plasmodium falciparum* typically harbour multiple haploid clones. Different parasite clones may vary significantly in immunogenicity, immune-avoidance mechanisms, susceptibility to drugs, and transmissibility by different *Anopheles* mosquito vector species [1]–[3]. The polyclonality and diversity of malarial infections together present a major barrier to vaccine development [4], [5]. The different parasite genotypes present in a single infection can be identified by analysis of polymorphic genetic loci, such as the merozoite surface protein genes *msp1* and *msp2*, amplified from peripheral blood samples of infected individuals [3], [6]. The apparent number of clones observed in any single infection thus depends on the diversity [in that individual) of the polymorphic marker used for the analysis, and the relative abundance of rare clones, which often fail to be detected among PCR products derived from numerically dominant clones. However, minority clones are of clinical interest as they may harbour genes conferring drug resistance and thus be selected by treatment, causing therapeutic failure [7], [8], or express antigenic variants unaffected by vaccine-elicited immunity [9].

Studies of the multiplicity of malarial infections have examined associations with the course and severity of infection, drug sensitivity, age, geographic origin, gametocyte production and infectivity to mosquitoes [10]–[19]. However, the most frequent application of clone analysis in malaria infections is in so-called “PCR correction” of recurrent infections in clinical trials of anti-malarial therapy. This can be confounded by the selective emergence after antimalarial treatment of minority clones in the pre-treatment parasite population [20]. Commonly used PCR methods share the limitation that such minority clones are under-represented or may be absent among assay products. This leads to mis-classification of PCR data in clinical trials, and underestimation of the extent of allelic polymorphism in any given infection [8], [21]–[24].

An alternative approach to investigating polyclonality in *P. falciparum* infections is adaptation of patient isolates to culture, and use of cloning and molecular genotyping techniques to analyse multiplicity *in vitro*. However, a recent study by Nsobya *et al.* [25], using isolates from Ugandan malaria patients propagated *in vitro*, demonstrated that there are significant inter-genotype differences in the ability to survive in even short-term cultures, leading to a rapid skewing of the parasite population *in vitro*, and an overall loss of complexity. Thus techniques which avoid expansion of parasite material, whether by polymerase chain reaction or by *in vitro* expansion, are more likely to preserve the complexity and relative abundance of different genotypes in patient isolates of interest.

The recent development of new generation direct sequencing technologies, capable of elucidating whole-genome data from relatively small biological samples, provides a potential new approach to investigate polyclonality in malaria infections. These technologies fractionate DNA samples into random end-tagged fragments of a uniform size, which are amplified *in situ* on a solid matrix, and then record the sequence of base addition to each growing amplicon. This produces a large number of short, but “massively” parallel sequence (MPS) reads which permit assembly of a partial or full genome provided an established reference genome sequence is available, and sufficient depth (number of reads at each nucleotide position) and breadth (proportion of the genome amplified and sequenced) of coverage are achieved. The single molecule sequencing approach of MPS technologies ensures that each sequence read (or pair of sequence reads if both ends of the molecule are sequenced) is essentially a haplotype, providing great scope for the characterisation of polyclonal infections. Using MPS, it is now possible to derive genome sequence data from a small volume of material from any organism of interest. Although assembly of extended genomic sequences is facilitated by the use of existing reference sequence, *de novo* or reference-free approaches are becoming more widely used [26]. MPS is now being assessed as a method to examine genome-wide polymorphism in *P. falciparum*, using both parasites cultured *in vitro* and material taken directly from the peripheral blood of people infected with the parasite [27]. However, the AT-rich genome of malaria parasites poses particular challenges for this approach to genome assembly [28], and thus it is unclear how well MPS will perform in analysis of *P. falciparum* taken directly from patients, particularly as natural infections commonly carry multiple clones with distinct genotypes at polymorphic loci.

In this study we use MPS to derive genome-level sequence data for five *P. falciparum* parasite isolates prepared directly from peripheral blood of four malaria patients, after minimal or no amplification of the parasite genome [28]. To evaluate the fidelity of the sequence data generated, and its utility for studies of genomic variation, we first examined structural differences among our isolates by global scanning for copy number variants (CNV). We then assessed sequence diversity at known polymorphic sites among six genes considered to be under strong selective pressure from antimalarial therapy, and among full-length mitochondrial sequences derived from each isolate. Finally, a genome-wide analysis of multiplicity was performed using other loci, selected empirically, which provided robust estimates of genotype multiplicity in each patient. These results were compared to conventional assessments of polyclonality using polymorphic loci encoding merozoite surface antigens.

## Methods

### Sample collection

Samples (OX001, OX003, OX005A, OX005B, OX006) were collected from returning travellers attending the Hospital for Tropical Diseases (HTD), or a referring hospital, with malaria symptoms, who were diagnosed positive by malaria films examined in the Department of Clinical Parasitology, and who gave written consent to have their blood samples analysed under a protocol approved by the University College London Hospitals Research Ethics Committee (project ref no. 07/0055). A minimum parasite density of 1% of infected erythrocytes was set as a cut-off to ensure DNA preparations included a high parasite to human white blood cell ratio.

### Processing of blood samples

EDTA blood remaining after diagnosis (2–4 mL) was prepared for genomic DNA extraction by removal of human white blood cells within four hours of venesection. Briefly, blood was diluted two to three-fold in phosphate-buffered saline, pH 7.2 (PBS), layered on to a 2 ml cushion of Lymphoprep® (Axis-Shield, UK), and centrifuged for 30 minutes at 4,000 g in a bench-top centrifuge at room temperature. Upper layers, including leukocytes, were removed, and the erythrocyte pellet was washed once in PBS and re-suspended to a total volume of 5 ml with PBS. The erythrocyte suspension was passed once through a Plasmodipu™ filter (Euro-Diagnostica), resuspended in 5 ml PBS and pelleted at 4000 g for 10 minutes. The erythrocyte pellet was stored frozen at −20°C, pending DNA extraction using the Qiagen DNA Blood Maxi (Qiagen, Crawley, UK) extraction kit.

Purified DNA samples were taken to the Wellcome Trust Sanger Institute (WTSI) malaria labs and prepared for MPS sequencing as described elsewhere [27], [28]. In parallel, each DNA sample was analysed for multiplicity using a standard nested PCR method to amplify polymorphic regions of *pfmsp1* and *pfmsp2* [6]. Amplicons were fractionated on agarose gels.

### Sequence Analysis

All samples underwent whole genome sequencing on the Illumina Genome Analyzer II platform, as previously described [27]. Briefly, paired 54- or 76-base pair sequence reads were generated on DNA fragments of 200–300bp. The fastq files for each lane of sequence data for each isolate were mapped onto the *P. falciparum* 3D7 reference genome V2.1.5 (ftp://ftp.sanger.ac.uk/pub/pathogens/Plasmodium/falciparum/3D7/3D7.latest_version/) using the Burrows-Wheeler Aligner (http://bio-bwa.sourceforge.net), resulting in *bam* files that can be analysed using SAMtools/BCFtools (http://samtools.sourceforge.net). Using this toolkit, we excluded reads of poor mapping quality, and derived a list of SNP (and small insertions and deletions (indels)) based on uniquely mapping reads and acceptable levels of coverage (minimum 10, max. 2000). In addition we applied a filter to rule out error-prone variant calls, based on a pseudo Phred Q-score, where a Q30/60 refers to one error per one-thousand/million bases. We used a threshold Q30, and the sensitivity of the estimated total number of SNP (and indels) to this score is shown in Table 1. Using this approach we identified SNP in the mitochondria genome (5.97 kb), and used tabulations of coverage across all SNP positions to call nucleotides. All raw sequence data (fastq format) used here are publicly available as fastq files, and as “Open Access” samples (prefix PL) on the WTSI Malaria Programme web-site [29].

**Table 1:** Summary of sequence data, and numbers of potential SNP and indels, relative to 3D7 reference sequence.

Isolate	Read length	Lanes	PE reads	Cover. All, >0	Cover.All, >0	Cover.	% genome	% genome	Q30	Q30	Q20	Q20	Q60	Q60
			per lane (millions)	Median (mean)	Nuclear median (mean)	Mito Median (mean)	Cover. >0	Cover. >4	SNP (Indels)	% coding (unique) regions	SNP (Indels)	% coding (unique) regions	SNP (Indels)	% coding (unique) regions
OX001	54	2	14.4	3, (11, 16)	3, 9 (11, 16)	1071 (1158)	68.2	45.4	27943 (1043)	69.2 (70.0)	29521 (1123)	68.5 (69.1)	24167 (785)	71.0 (72.1)
OX003	54	2	13.9	6, 9 (11, 14)	6, 9 (11, 14)	939 (895)	80.7	54.5	27093 (1546)	65.4 (70.1)	28538 (1644)	64.5 (69.1)	23699 (1249)	67.6 (70.2)
OX005A	76	2	32.6	90, 91 (98, 101)	90, 91 (97, 100)	1244 (1150)	97.5	95.5	48329 (25763)	31.4 (74.5)	50442 (26419)	31.3 (74.0)	43059 (24239)	31.9 (76.5)
OX005B	76	1	30.0	126, 127 (115, 118)	125, 127 (114, 117)	1544 (1478)	97.5	96.1	43753 (22624)	33.8 (76.7)	46258 (23208)	33.4 (75.6)	37010 (21180)	34.5 (79.8)
OX006	76	1	25.1	122, 123 (115, 117)	122, 123 (115, 117)	1526 (1450)	98.0	96.7	42985 (25580)	32.5 (78.1)	45863 (26687)	32.3 (77.1)	34307 (23834)	32.5 (81.1)

PE =  paired end; Cover.  =  coverage; all refers to all positions; >0 refers to those positions with non-zero coverage; Mito =  mitochondrial genome; unique  = % of sliding 50-mer windows around each position that are unique; indels =  insertions and deletions; Q20/30/60 equates to error rates of 1 in 100/1000/1000000 base pairs respectively.

### Identification of copy number variation

CNV, including deletions and sequence amplifications, may be identified using sequence coverage data. In particular, regions with little or no coverage may be deleted in some or all genomes in the infection, whilst those with coverage in excess of the average may be amplified. A list of small indels from the paired end mapping process with evidence from at least two isolates was constructed using SAM tools with the same Q30 threshold. In particular, small indels are detected by comparing the distance between mapped read pairs to the average insert size of the genomic library [30], [31]. For each isolate, we then calculated total coverage at positions in the sequence that were in coding regions and were classified as unique by sliding 50-mers across the reference genome. To further identify regions with CNVs we adopted a similar approach to Yoon *et al.* [32]. In particular, coverage data were normalised for GC content, and a Z-score statistic calculated using 100-bp windows by subtracting the mean of all windows and dividing by the standard deviation. These Z-scores were converted to p-values and we report any regions lower than a 10^−6^ threshold. Regions of interest were examined graphically using LookSeq software [33] (available at http://lookseq.sanger.ac.uk/lookseq2/index.html).

### Analysis of drug resistance-associated loci

Illumina nucleotide calls were extracted from genome read data at each position for the following six loci, implicated in resistance to artemisinins or other antimalarials: (i) *pfatpase6* (PFA0310c, chr 1: 265 447-269 412); (ii) *pfmrp1* (PFA0590w, chr 1: 465 875–471 344); (iii) *pfmdr1* (PFE1150w, chr 5: 957 885-962 144); (iv) *pfcrt* (MAL7P1.27, chr 7: 458 990–461 216); (v) *pfmrp2* (PFL1410c, chr 12: 1 192 877–1 199 204); *pfnhe1* (P13_0019, chr 13: 170 076–175 991). Sequence from each locus was converted to fasta format after manual removal of introns, and compared to sequence from the other isolates, and the 3D7 reference genome, by alignment in Clustal W (http://www.ebi.ac.uk/Tools). At these loci, coverage depth was typically 40 to 100 reads; multiplicity at a specific site was often observed, indicated by more than one nucleotide being called; a low threshold (2 calls) was set for retaining minor variants in the analysis, irrespective of the total read coverage at that position. Coverage breadth was excellent, being close to 100% for each resistance-associated locus.

### Genome-wide screen for minimal multiplicity

For each of our test isolates we inferred the minimal multiplicity by considering the number of distinct haplotypes formed by combinations of three SNP on single or paired reads. We removed any haplotypes that had a frequency of one, and ignored those sets of haplotypes with total frequency (coverage) less than 10, or were located in genomic regions that were not unique (defined using 50-mers, see above).

## Results

### Patients

Between December 2008 and December 2009, subjects gave consent for genetic studies on parasite isolates derived from the diagnostic blood samples taken as part of their routine care. Five DNA samples from four of these volunteers were deemed suitable for taking into the Illumina DNA sequencing pipeline. For one patient (OX005), two sequential samples, taken at 0 hours (i.e. pre-treatment) and 9 hours after treatment, were analysed in parallel. All patients were successfully treated and recovered from malaria.

#### OX001

This patient was a 53-year old Ghanaian who had lived in the UK all their adult life and presented with symptoms of acute malaria after a visit to Accra and the surrounding countryside for a period of six weeks. Having started to feel unwell in Ghana, the patient reported taking one (unidentified) tablet which was bought from a street trader, but reported no other treatment, and did not use chemoprophylaxis.

#### OX003

This 26-year old Zimbabwean-born Caucasian patient had lived in the UK for four years and reported having malaria 3 times before moving to the UK. The patient presented with circulating *P. falciparum* trophozoites and schizonts at a parasitaemia of 1.3% following a visit to Mozambique (Mapute/Tete) for a period of three weeks, during which no chemoprophylaxis or treatment was taken. A single pre-treatment blood sample was analysed.

#### OX005

Following a visit to family in Ghana, this 49-year-old Ghanaian-born UK resident (of 22 years) presented with severe malaria and a peripheral *P. falciparum* parasitaemia of 3.0% (details presented in reference 34). Two sequential samples, F54840 and F55564, taken prior to treatment and 8 hours later (4 to 5 hours after commencement of quinine treatment), respectively, were analysed.

#### OX006

This case is also presented in detail in reference 34. Briefly, a 39-year-old European, resident in the UK and with no previous malaria episodes, presented with severe *P. falciparum* hyperparasitaemia (39%) after returning from 10 days in Kenya. One sample, taken approximately 2 hours after commencing intra-venous artesunate therapy, was analysed.

Data for all isolates described are publicly available via the MapSeq portal at the WTSI (http://www.sanger.ac.uk/MapSeq/) and have the following Sanger identifiers within the PF14 study folder: OX001 = PL0001, OX003 = PL0002, OX005 = PL0004, OX006 = PL0005.

### Evidence of variation in gene copy number in patient isolates

**Figure 1:**
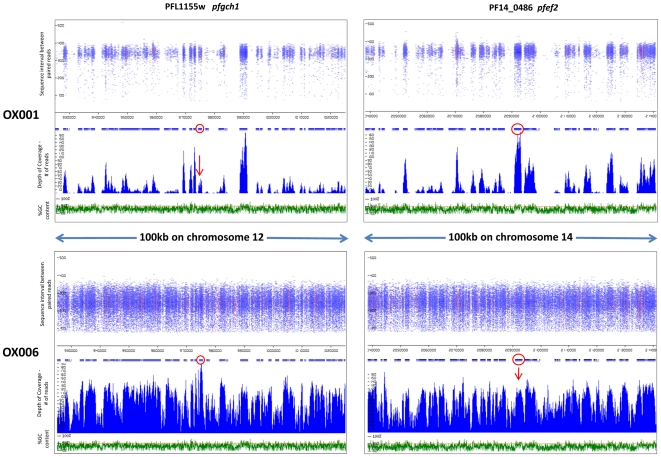
Identifying amplifications as areas of high MPS coverage. Length of sequence intervals between paired reads (nt) and coverage (read frequency) are plotted against chromosome position and %GC content for two loci in isolates OX001 and OX006. *pfgh1* displays high coverage consistent with amplification in OX006, but not OX001. *pfef2* displays high coverage consistent with amplification in OX001, but not OX006. Loci of interest (red circles) are shown within100 km of genomic context. Red colouring within the read pile-ups signify polymorphic sites within a read in which a non-reference allele is present (i.e. SNP).

Read coverage, sequence intervals between read pairs and GC content were examined around loci of interest, identified as described in Methods. Two examples of gene amplification are presented in Figure 1, in isolates OX001 and OX006. The richness of data clearly differs between these two isolates, as the former was analysed using an earlier format of the MPS procedure which utilised 54nt reads, whereas 76nt reads were used in analysis of OX006 (Table 1; Fig. 1). The *pfgch1* locus, which has previously been identified as undergoing amplification in Southeast Asian parasite isolates [35], showed evidence of excess coverage in isolate OX006, but not in isolate OX001 (Fig. 1), nor in the other 4 isolates (data not shown). The PF14_0486 locus (*pfef2*) encodes an EF2 translation elongation factor related to Ef2b isoforms of metazoan eukaryotes, with 65% amino acid sequence identity to *Drosophila melanogaster* Ef2b (accession number AY075481). *pfef2* displayed high coverage consistent with amplification in isolate OX001, but not in OX006 (Fig. 1).

**Figure 2:**
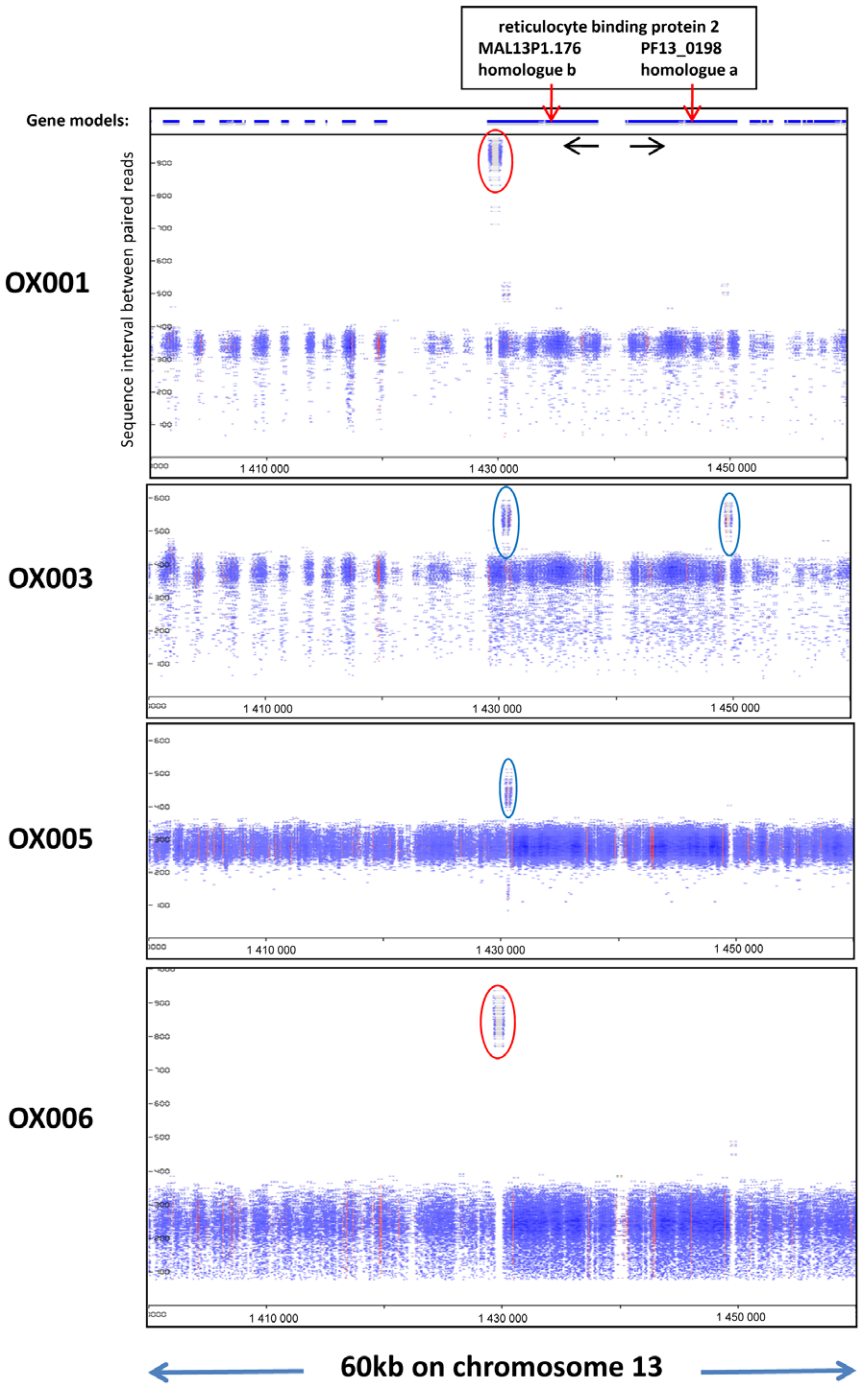
Deletions in *pfrbp2* homologues a and b appear as areas with inflated sequence intervals in four isolates. Two isoforms of RBP2 are encoded by adjacent genes on chromosome 13, arranged head to head and transcribed in opposite directions. 60 kb around these genes are depicted, for four isolates. Loci of interest (red arrows) have either a ∼600 bp deletion in the carboxy-terminal serine-rich domain of homologue b (red elipses), or a ∼2–300 bp deletion in the low-complexity protein domain immediately upstream in both genes (blue elipses). Y-axis depicts sequence interval between paired reads. X-axis depicts nucleotide coordinates along the chromosome, as in Figure 1.

Evidence of deletion of genomic sequences, defined with respect to the 3D7 reference genome sequence, was found at numerous loci across the genome in each isolate. Figure 2 presents pile-up graphics [33] for two isoforms of *pfrbp2* encoded on chromosome 13 (MAL13P1.176; PF13_0198). The data clearly distinguish between two previously described deletions that are common towards the carboxy terminus of MAL13P1.176, encoding the b-homologue of *pfrbp2*: OX001 and OX005 are to seen to have apparent read intervals of ∼950nt over this domain, which is approximately 600 bp longer than the average read length in that chromosome region (Fig. 2). This fits well with the expected 587 bp deletion in the serine-rich domain, which was recently shown to be common across Africa [35]. In contrast, OX003 and OX004 show evidence of a smaller deletion in the low complexity domain immediately upstream of the serine-rich region. Interestingly, this is also seen at the C-terminus of the a-homologue (PF13_0198), which lacks the serine-rich domain and does not exhibit the deletion-polymorphism characteristic of the adjacent b-homologue. Among other loci displaying similar evidence of intra-genic deletion in one or more of our clinical isolates was the conserved *Plasmodium* protein (unknown function) PF14_0226 in which the 12 amino acid repeat motif S(or K or W)TLKEKKNEMNV occurs in 7 tandem repeats in 3D7, but typically occurred as 2 to 5 repeats in MPS-derived genome sequences from our isolates. Verification that this observation is not due to ambiguous assembly of repeats could be accomplished simply by amplification and sequencing of this region of the gene.

**Figure 3:**
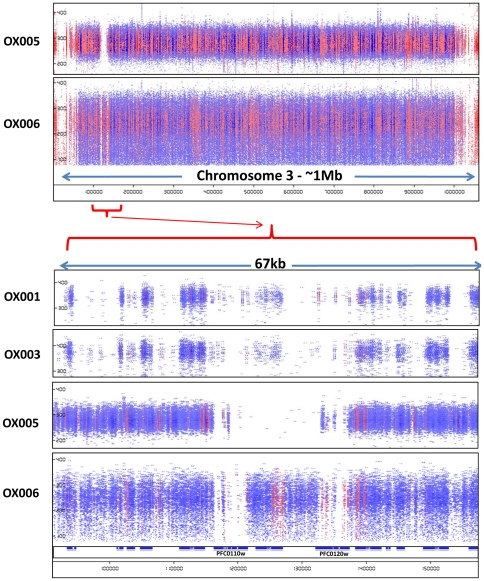
Evidence of a major deletion at the right end of chromosome 3 in isolate OX005. Paired reads across the whole of chromosome 3 are presented in pile-up view for two isolates, OX005 and OX006 (upper panel). Y-axis depicts sequence interval between paired reads, and X-axis gives chromosome coordinates as in Figures 1 and 2. A detailed view of ∼70 kb around the *clag3.2* and *clag3.1* loci is also shown for 4 isolates (lower panel). The locus between PFC0110w and PFC0120w is a degenerate *var* gene lacking a full-length ORF in 3D7 and other parasite sequences in the available databases.

Of particular note was occasional evidence of larger chromosomal deletions, such as that encompassing one or both of the loci encoding Rh1/Clag3.1 and Rh1/Clag3.2 (PF0110w and PF0120w) at the left end of chromosome 3. In isolates OX005 and OX005A, both genes and the chromosomal region between them appeared to be deleted, totalling over 20 kb of missing chromosome sequence compared to the reference. This is consistent with reports of variation in the size of this chromosome estimated using pulse-field gel electrophoresis, with 3D7 carrying a larger 3^rd^ chromosome than other isolates tested in one study [36]. Thus the duplication of the *clag3* locus described in laboratory lines [37] was not readily identified in MPS sequence data of our wild isolates. Interestingly, the PfEMP1 pseudogene between the two *clag3* loci in 3D7 was identified by MPS in some, but not all of our isolates (Fig. 3).

### Sequence polymorphism in known loci under drug selection

In order to evaluate the potential of MPS analysis using parasite material taken directly from treated malaria patients for phenotype-genotype association studies, we derived full length data for 6 loci known or considered likely to be under selective pressure from antimalarial drugs (see Methods). Data for all 5 isolates and all 6 loci are presented in Table 2. In each of these genes, known polymorphisms were identified. Both the reference and variant alleles were present in at least one gene from each of the isolates OX001, OX003 and OX006. This indicates that these were polyclonal infections, and that multiple alleles of these genes were present. Both isolates from patient OX005 appeared monoclonal (and identical) at these loci, except for apparent polyclonality in the repetitive “hinge” region of *pfmdr1* and in a three amino-acid repeat region of *pfmrp2*. In both loci, this may conceivably be caused by ambiguous assembly of highly-repetitive sequences, but we cannot rule out true polyclonality in both these isolates (see further analysis below).

**Table 2:** Amino acids encoded at polymorphic codons of 6 *P. falciparum* loci likely to be under drug selection.

Isolate	Pfatpase6 PFA0310c			Pfmrp1 PFA0590w		Pfmdr1 PFE1150w					Pfcrt MAL7P1.27			Pfmrp2 PFL1410c									Pfnhe1 P13_0019				
CODONS:	431	569	639	876	1466	86	184	496	649–654	1246	72–76	220	271	199	235–40–42	350	709	714	796	1527	1531	1373	173	203–4	878	950	1557
3D7 REF	E	N	G	I	K	N	Y	T	NDDNNN	D	CVMNK	A	Q	L	YQQ	T	Q	K	S	S	L	H	V	SD	T	V	F
OX001 Ghana	E	N	G	I	K	YN	F	T	NDDNNN	YD	CVMNK	A	Q	LV	YQQ	PT	QK	K	S	-	-	N	V	SD	T	V	S
OX003 Mozambique	E	K	G	I	K	N	F	I	NDDNNN DDNNNN	D	CVIET	S	E	V	YQQ	T	Q	I	AS	T	VI	H	A	SD	T	G	S
OX005A Ghana	E	N	G	I	K	N	F	T	NDNDNN YNDNNN	D	CVMNK	A	Q	V	YQQ YEE	T	Q	I	S	T	I	H	A	FY	I	V	S
OX005B Ghana	E	N	G	I	K	N	F	T	NDDDNN NNDNNN	D	CVMNK	A	Q	V	YQQ NEE	T	Q	I	S	T	I	H	A	FD	I	V	S
OX006 Kenya	K	N	D	VI	R	YN	YF	T	NNNNDD NDDNNN	YD	CVIET	S	E	V	YQQ NQE	T	Q	I	S	ST	LI	H	V	SD	T	G	S

Genome sequence data was generated on the Solexa Illumina platform as described in Materials and Methods. Sequences aligning with the 6 loci shown in the reference sequence for *P. falciparum* (laboratory clone 3D7) were extracted (with quality criteria stated in the Methods), converted to FASTA format, translated and aligned in Clustal W. Sites exhibiting polymorphism among the 5 isolates are shown.

Shading: non-synonymous substitution relative to the reference sequence.

Multiplicity: where more than one base was called at any one position, the encoded amino acid with the most calls is displayed above. Haplotypes cannot be inferred by these data – for example any or all combinations of YFY, YFD, NFY or NFD may exist for *pfmdr1* codons 86, 184 and 1246 in patient OX001.

Recent data suggest that the two amino acid changes in the *P. falciparum* ABC transporter MRP1 observed in isolate OX006 are associated with artemisinin and anti-folate resistance in African studies [38], [39], and it is possible that the response to artesunate-treatment of the parasites in this individual may have been delayed [34]. Examination of the *pfmrp1* sequence at codon 876 in later follow-up blood samples from this patient may be instructive, as this locus exhibited mixed wild-type and mutant alleles in the immediately post-treatment sample analysed here. The data presented on *pfmrp2* represents the first full-length analyses of this gene in wild parasites; 8 non-synonymous codons were found in addition to the amino-acid repeat polymorphism at codons 235, 240 and 242. In *pfmdr1*, which encodes the best characterised transporter protein in *P. falciparum*, PgH1, we encountered the expected polymorphisms at codons 86, 184, 649–654 and 1246. Unexpectedly, our Mozambique isolate also harboured a novel non-synonymous substitution, T496I. This region of the locus is not usually analysed in sequencing studies, and there are no data to suggest whether or not this substitution is relevant to parasite responses to antimalarial treatment.

The less stringent call threshold for SNP used in our analysis of drug-resistance associated loci permitted identification of low frequency amino acid variants at expected positions: for example in the *pfmdr1* locus of isolate OX001 at codon 86 there were 46 MPS “calls” for T at nucleotide 256, encoding the resistance-associated tyrosine at this position, and 6 calls for the wild-type nucleotide A, encoding asparagine. Similarly, at codon 1246 there were 42 calls for T at nucleotide 3756, encoding a resistance-associated tyrosine, and 4 calls for G, encoding aspartic acid. Thus, as these low frequency calls generated sequences consistent with previous studies of the locus, we conclude that they are *bona fide* results, and so caution should be exercised in setting high call thresholds in MPS studies of potentially polyclonal *P. falciparum* isolates. This approach also readily identified substitutions of interest in the *pfdhfr* (PFD0830w) and *pfdhps* (PF08_0095) loci which are known to modulate parasite sensitivity to the anti-folate drugs (Table 3). All isolates harboured the “triple mutant” haplotype IRNI at codons 51, 59, 108 and 164 of *pfdhfr*, but three different haplotypes of *pfdhps* were seen. Only the Kenyan isolate OX006 harboured the *pfdhps* 540E mutation associated with high rates of treatment failure with sulphadoxine-pyramethamine, and known to be much more common among travellers returning with malaria from east Africa [40].

**Table 3:** SNP and inferred haplotypes in *pfdhfr* and *pfdhps* loci.

	Isolate ID				
**Chromosome coordinate Gene ID**	OX001	OX003	OX005A	OX005B	OX006
MAL4:755220 PFD0830w	T	T	T	T	T
MAL4:755243 PFD0830w	C	C	C	C	C
MAL4:755391 PFD0830w	A	A	A	A	A
MAL4:755558 PFD0830w	A	A	A	A	A
**DHFR haplotype codons 51_59_108_164**	**IRNI**	**IRNI**	**IRNI**	**IRNI**	**IRNI**
MAL8:550802 PF08_0095	T	T	G	G	T
MAL8:550806 PF08_0095	G	G	G	G	G
MAL8:551114 PF08_0095	A	G	A	A	G
MAL8:551238 PF08_0095	C	C	C	C	C
**DHPS haplotype codons 436_437_540_581**	**SGKA**	**SGKA**	**AGKA**	**AGKA**	**SGEA**

In summary, specific analysis from our MPS data of a series of loci implicated in drug response identified expected polymorphisms in each gene, strongly suggested polyclonality in most if not all isolates, and suggested setting of low call thresholds can assist in identifying low abundance sequences. The analysis identified previously unknown substitutions in *pfmdr1* and *pfmrp2* that now require evaluation as possible markers of parasite response to antimalarial drugs.

### Polymorphism in mitochondrial genome sequences

Polymorphic sites in the 5.97 kb mitochondrial genome were derived for each sample by the identification of nucleotides that differed to the reference sequence. As expected the mitochondria had at least ten-fold more coverage than the nuclear genome (Table 1). The full mitochondrial sequence was generated for each patient isolate, and compared against the panel of 32 polymorphic sites by Joy *et al.* [41] (Table 3). The parasite isolates were in each case almost identical to the reference sequence. Three polymorphisms were found, and two corresponded to sites previously identified by Joy and colleagues. These results indicate that MPS sequencing data derived from uncloned and uncultured parasite material is of a sufficiently high quality for use in phylogenetic studies based on variation in the parasite mitochondrial genome.

### Clonal multiplicity estimated from conventional pfmsp2 genotyping

Nested PCR analysis of the *pfmsp1* and *pfmsp2* locus was undertaken to estimate the polyclonality of each isolate using conventional methods. The results are presented in Table 4. All isolates except OX005A harboured polyclonal infections as evidenced by analysis of *pfmsp1* and *pfmsp2* alleles, with five, four, one, two and six distinct alleles, respectively. It was observed that isolate OX005B, taken 9 hours after initiation of quinine therapy, harboured an additional *P. falciparum* genotype not present in isolate OX005A, taken prior to the first dose.

**Table 4:** Polymorphic nucleotide positions in MPS-derived *P. falciparum* mitochondrial genomes.

Mitochondrial genome coordinates																																
NT coord: Joy et al. 30	74	204	701	766	776	837	964	1260	1284	1362	1371	1634	1687	1696	1754	1780	1938	2179	2387	2495	2645	3010	3070	3517	3558	3729	3858	3966	4184	4718	4720	4956
Ref state (version 2.1.4)	A	C	**ATAT**	A	C	T	T	G	G	G	G	A	A	G	T	T	G	T	G	G	T	T	G	T	C	C	T	A	C	A	A	T
Coding Gene				**cox3**														**cox1**						**cytb**								
**ISOLATES**																																
OX001 Ghana	A	C	T	A	C	T	T	G	G	G	G	A	A	**A**	T	T	G	T	G	G	T	T	G	T	C	C	T	A	C	A	A	**C**
OX003 Mozambique	A	C	T	A	C	T	T	G	G	G	G	A	A	G	T	T	G	T	**A**	G	T	T	G	T	C	C	T	A	C	A	A	**C**
OX005A Ghana	A	C	T	A	C	T	T	G	G	G	G	A	A	G	T	T	G	T	G	G	T	T	G	T	C	C	T	A	C	A	A	**C**
OX005B Ghana	A	C	T	A	C	T	T	G	G	G	G	A	A	G	T	T	G	T	G	G	T	T	G	T	C	C	T	A	C	A	A	**C**
OX006 Kenya	A	C	T	A	C	T	T	G	G	G	G	A	A	**A**	T	T	G	T	G	G	T	T	G	T	C	C	T	A	C	A	A	**C**

Row 1: Nucleotide coordinates from ref. 30;

Row 2: additional “ATAT” insert at position 701 is not present in reference sequence.

Row 3: intersection of polymorphic loci with protein-coding genes

### Genome-wide multiplicity from MPS data

Multiplicity was estimated only for loci in which 3 single-nucleotide polymorphisms (SNP) occurred in close proximity, using two different approaches. The first analysis identified loci where three SNP were found on a single read of 54 or 76 bp (Table 1). In the second analysis, tri-SNP loci were deployed in which one of the SNP occurred on an independent sequence read adjacent to the read with the other two SNP (a "2+1” SNP trio). This latter approach could be seen as potentially the more robust, as false 3-SNP haplotypes are less likely to be generated by errors in two adjacent reads, than in a single poor quality sequence read. The first analysis identified 139 polymorphic tri-SNP loci that indicated a multiplicity of 5 genotypes or more across the whole genome in at least one isolate. The majority of these loci did not map to annotated genes in the reference genome, and very few loci displayed high apparent multiplicity in more than one isolate. However, there were clusters of adjacent loci in the same isolate giving high multiplicity estimates, and these suggest the methods deployed were correctly identifying highly polymorphic genomic regions. In the second more stringent analysis, using “2+1” SNP trios across paired reads, 52 tri-SNP haplotypes were identified. Multiplicity estimates derived from both methods are compared to those derived from PCR data in Table 5.

**Table 5:** Clonal multiplicity estimated from polymorphic amplicon sizes in *pfmsp1* and *pfmsp2* PCR assays compared to estimates from MPS analysis.

Number of alleles seen*		OX001 Ghana	OX003 Mozambique	OX005A Ghana	OX005B Ghana	OX006 Kenya
**MSP-1**						
K1		2	1	2	2	2
MAD20		0	2	1	1	1
RO33		2	1	0	0	1
**MSP-2**						
FC27		2	2	1	1	3
IC/3D7		3	1	0	1	1
Minimum number of genotypes from PCR analysis		**5**	**4**	**3**	**3**	**4**
Minimum number of genotypes from MPS analysis **	Method 1	**6**	**5**	**6**	**7**	**7**
	Method 2	4	4	3	-	5

*Each of the three allelic families occur in a mutually exclusive manner in a single *msp1* gene; similarly for the two allelic families of *msp1*. Thus the minimum number of genotypes is taken as the larger of the allele totals for the two genes.

**Highest minimum estimates of haplotype multiplicity (> = 3) are shown for each isolate. Method 2 did not identify high multiplicity loci in isolate OX005B.

Estimates of clonal multiplicity derived from MPS analysis were similar, but slightly higher than those obtained from analysis of *msp1* and *msp2* alleles by PCR. Therefore, we examined the data for evidence that the same loci were generating high multiplicity estimates in more than one isolate, and thus might be generally useful tools for clonal multiplicity studies, and also looked for overlap between the two methods used. Two highly repetitive loci contributed to elevated estimates of multiplicity in more than one isolate, but these were only seen when using the first (less stringent) method. The first of these, on chromosome 7, lies in the distal sub-telomeric repeat region, and thus is unlikely to provide an accurate estimate of clone multiplicity. The second locus, within the gene P10_0265, which encodes a conserved *Plasmodium* protein of unknown function, is within a region of open reading frame that is highly repetitive. A tri-nucleotide repeat (CAA/CAG) occurs 67 times in the 3D7 reference sequence, encoding an extensive poly-glutamine tract. Analysis of read-length at this locus [33] confirmed apparent high multiplicity around these repeat sequences in each isolate, but it is likely this is the result of mapping inconsistencies due to repeat overlaps. (Confirmation of the utility of this locus for estimating multiplicity would require empirical size comparison of appropriate PCR amplicons from different isolates.) These findings support the use of the more stringent “2+1” SNP trio approach for identification of multiplicity at polymorphic loci.

## Discussion

In this study, we have demonstrated that MPS analysis of the genomes of *P. falciparum* parasites isolated directly from infected malaria patients generates high quality data that can be used to identify known and unknown polymorphisms in loci of interest. In particular we demonstrated the utility of this approach to identify genomic sites where both previously described and novel CNV occurred. We were also able to elucidate, directly from MPS data, drug-resistance associated haplotypes of candidate loci, and succeeded in generating high quality mitochondrial genome sequence data suitable for phylogenetic studies. Some of the polymorphisms identified in drug resistance-associated loci and mitochondrial genomes have been previously described, but others were identified here for the first time and can now be investigated in more detail.

The main objective of the study was to investigate the utility of MPS analysis for estimating polyclonality in natural parasite isolates, as accurate estimation of clone multiplicity is important for studies of drug efficacy and parasite population diversity [42], [43]. Using a genome-wide approach, reproducible estimates of clonal multiplicity in our clinical isolates were obtained, and these were similar to estimates of clonal multiplicity estimated by standard methods based on well-characterised size and sequence polymorphisms in the *pfmsp1* and *pfmsp2* genes. Thus we have confirmed by two completely independent methods a high multiplicity in all of our patients. Multiplicity was also seen in our analysis of drug resistance-associated loci, but as expected of loci under strong directional selection, in each case only two alleles at each position were observed, reflecting lower overall diversity in these genes.

How does a traveller, in many cases only briefly exposed to malaria infection risk, come to harbour multiple parasite clones? Previous studies have quantified the risk to be one *P. falciparum* infection per 1.36×10^5^ UK traveller weeks in an endemic area. This suggests the probability of a single individual receiving more than one infective bite per week is approximately one in one hundred thousand [44], [45]. However, as these studies were based on Thai data, it is likely multiple inoculations are more likely in the African locations where our patients acquired their *P. falciparum* infections: the precise chance that an individual received multiple inoculations will differ depending on the endemicity of infection in the area visited, microheterogeneity in mosquito biting and infection rates, use of and adherence to bite prevention and chemoprophylaxis and the fact that multiple areas of differing risk may have been visited by the same traveller [13]. Nevertheless, the likelihood is that in most imported cases of *P. falciparum* infection, the entire clonal repertoire entered the host in a single bite. Thus our patients' genetically complex parasite infections are expected to reflect the complexity circulating in endemic area human populations; further, as mixed gametocyte genotypes have been shown to circulate in African studies [46], [47], recombinant zygotes, heterozygous at polymorphic loci, are expected to be common. Thus an infection with multiplicity 6 must be the product of at least 3 genetically unrelated oocysts, each of which was heterozygous. This oocyst diversity could have built up in the mosquito over a few days as, once infected, mosquitoes are known to continue feeding on subsequent nights [48]. A single female *Anopheles* may thus have several developing oocysts at slightly different stages of maturity. This would imply that each sporozoite inoculum may be presenting in a single event the progeny of several blood meals in which the mosquito was infected with a variety of parasite genotypes.

MPS data are well suited to long-range investigation of copy number variations in both coding and non-coding genomic DNA. Using tools freely available on-line at the WTSI, we were able to identify in our MPS data, almost to the exact base-pair, a previously described 587 bp deletion in the b-homologue of *pfrbp2* [49]. The evidence for genomic amplification of *pfef2* (PF14_0486) in OX001 is of interest. Although this nuclear locus encodes a eukaryotic-type cytoplasmic translation elongation factor similar to those of *Drosophila*, prokaryotic-type EF genes in the *Plasmodium* apicoplast genome encode a group of proteins known to be the parasite targets of antibacterial drugs such as clindamycin and doxycycline [50]. It is unknown whether amplification of cytoplasmic elongation factor genes such as *pfef2* might play a role in parasite drug responses.

An unexpected finding was the evidence suggesting two rhopH1/clag3 genes on chromosome 3, and the var pseudo gene located between them, were deleted in one of our isolates. Paired read data from other isolates was also suggestive of partial or full deletion of the clag sequences. A possible explanation for this is that mistakes in the assembly of MPS end-reads were made due to the fact that these genes belong to a multi-gene family dispersed across the genome at several loci. However, large areas of null read coverage were not common in our dataset, and other members of the clag gene family on chromosomes 2, 7 and 9 were examined and displayed similar or better read coverage than their immediate genomic context. Further alignment of GENBANK sequences for clag3.1 and clag3.2 loci from 3D7, FVO and 7G8 laboratory strains demonstrated remarkable conservation (>90% sequence identity at nucleotide level) among these loci. Therefore, extreme polymorphism at these loci is an unlikely explanation for poor read coverage. Given the observations of Kemp et al. that chromosome 3 can vary significantly in size on pulse-field electrophoresis [36] and the evidence of mutually exclusive expression of clag3.1 and clag3.2 proteins in different cultured lines derived from 3D7, suggesting functional redundancy [37], our data may be interpreted as a demonstration that *P. falciparum* can propagate perfectly well *in vivo* without one or both of the clag loci on chromosome 3. Unfortunately, genomic material from our patients was not prepared in such a way as to permit whole chromosome preparations for analysis on pulse-field electrophoresis, in order to verify this interpretation.

### Concluding remarks

This study provides proof-of-principle that MPS data from material obtained directly from malaria-infected *Homo sapiens* is suitable for a variety of genomic, phylogenetic, parasitological and clinical analyses. A great strength of MPS in general is the prospect of high-throughput analysis of a large number of genomes in parallel [27]; we have only examined a handful of genomes, and thus have not taken advantage of this aspect of the technology. However, an important feature of our analysis is that we have used well characterised clinical material with follow-up post-treatment DNA samples available, permitting further studies of any locus of interest identified in the pre-treatment isolate. Thus, we have been able to compare the pre-treatment genome sequence, for one of our patients, with the sequence of an isolate taken after 9 hours of antimalarial treatment. It follows that, in some settings, genomic-level follow-up studies of drug selection signals are eminently feasible.
